# Financial difficulties in breast cancer survivors with and without migration background in Germany—results from the prospective multicentre cohort study BRENDA II

**DOI:** 10.1007/s00520-022-07074-7

**Published:** 2022-05-04

**Authors:** N. Riccetti, R. Felberbaum, F. Flock, T. Kühn, E. Leinert, L. Schwentner, S. Singer, K. Taylor, A. Wöckel, W. Janni

**Affiliations:** 1 Institute of Medical Biostatistics, Epidemiology and Informatics, University Medical Centre, Johannes Gutenberg University Mainz, Mainz, Germany; 2 Department of Gynaecology and Obstetrics, Hospital Kempten, Kempten, Germany; 3 Department of Gynaecology and Obstetrics, Hospital Memmingen, Memmingen, Germany; 4 Department of Gynaecology and Obstetrics, Hospital Esslingen, Esslingen, Germany; 5 Department of Gynaecology and Obstetrics, University of Ulm, Ulm, Germany; 6 Department of Gynaecology and Obstetrics, University of Würzburg, Würzburg, Germany

**Keywords:** Neoplasm, Breast cancer, Migration, Financial burden, Psycho-oncology, Access to care

## Abstract

**Purpose:**

We aimed to explore the trajectory of financial difficulties among breast cancer survivors in the German health system and its association with migration background.

**Methods:**

In a multicentre prospective study, breast cancer survivors were approached four times (before surgery, before and after adjuvant therapy, five years after surgery) and asked about their migration history and financial difficulties.

Migrants were defined as born/resided outside Germany or having citizenship/nationality other than German. Financial difficulties were ascertained with the financial difficulties item of the European Organisation for Research and Treatment of Cancer Core Instrument (EORTC QLQ-C30) at each time-point (cut-off > 17). Financial difficulties were classified in trajectories: *always* (every time-point), *never* (no time-point), *initial* (first, not fourth), *delayed* (only fourth), and *acquired* (second and/or third, not first).

A logistic regression was conducted with the trajectories of financial difficulties as outcome and migration background as exposure. Age, trends in partnership status, and educational level were considered as confounders.

**Results:**

Of the 363 participants included, 49% reported financial difficulties at at least one time-point.

Financial difficulties were reported always by 7% of the participants, initially by 5%, delayed by 10%, and acquired by 21%.

Migrants were almost four times more likely to report delayed (odds ratio [OR] = 3.7; 95% confidence interval [CI] 1.3, 10.5) or acquired (OR = 3.6; 95% CI 1.6, 8.4) financial difficulties compared to non-migrant participants.

**Conclusion:**

Survivors with a migration background are more likely to suffer from financial difficulties, especially in later stages of the follow-up. A linguistically/culturally competent active enquiry about financial difficulties and information material regarding supporting services/insurances should be considered.

**Supplementary Information:**

The online version contains supplementary material available at 10.1007/s00520-022-07074-7.

## Introduction

Between 21 and 44% of patients with cancer and cancer survivors report financial difficulties throughout the disease [1–5]. These difficulties, if unrecognized and unsupported, could negatively impact the physical, psychological, and socio-economic status of the patients/survivors, leading to poorer access to health services (e.g. suboptimal adherence to therapy, lower participation to follow-up/testing), and therefore to poorer health status and health-related quality of life [1, 3, 6–14].

Financial difficulties were often related to the direct payment of medical costs: cancer survivors with high out-of-pocket payments were more likely to report problems paying medical bills compared to patients with cancer with low out-of-pocket payments and compared to their siblings without cancer [15]. For one cancer patient in four, medical costs were not affordable (26%) or the disease resulted in a financial debt (22%) that often lasted longer than one year after the diagnosis [1, 12]. Almost one patient in three (30%) and one in six (15%) reported to have used money from savings and retirement accounts, respectively, as a consequence of the disease [1].

Psychological aspects of financial difficulties, such as anxiety or fear of not being able to pay for medical costs or to become a burden for the family, were also reported [10, 16, 17]. Among patients with cancer, 48% reported elevated cost-related anxiety [16], and 21% of cancer survivors reported being worried about paying medical bills [10].

Most of the studies on financial difficulties among cancer survivors were conducted in countries with healthcare systems based on out-of-pocket payments or private insurances, different from and not comparable to the healthcare system in Germany [5, 18]. The German health system is based on a combination of mandatory public and private insurances; out-of-pocket payments are estimated to be 12% of all health-related costs [19]. Hence, financial difficulties among cancer survivors in Germany are less related to the direct costs of the treatment and more to the non-medical costs (e.g. transportation costs [5]) and to the inability to work. Work leave and return to work are key aspects for cancer care, both for the survivors and for the society [11, 20]. Due to the improvements in early detection and treatment and to the increase in retirement age, about half of cancer patients and survivors are of a working age [21]. The mean time absent from work among women with early-stage breast cancer is estimated to be 11.4 months, with approximately 36% of patients absent for more than one year and 12% for more than two [22, 23]. Furthermore, between 27 and 37% of cancer survivors do not return to work at all [20, 24]. Overall, survivors have a 40% higher risk of being unemployed compared to people without cancer [25]. This often leads to a reduction in the expected salary: for example, during the first year following the diagnosis, women with breast cancer experience a loss of more than a quarter of their earnings (27%) [26].

The presence of financial difficulties in patients with cancer and cancer survivors has been associated with younger age [1, 3], female gender [3], marital status [3], lower monthly net income [1], educational level [12], and self-reported worse health status [3].

In addition, the presence of financial difficulties has been associated with the ethnicity of the patients and survivors [1, 3, 12, 17]. African American and Asian American patients with cancer have reported higher financial difficulties than White American patients [3]. Black patients with cancer had a 63% higher risk to experience financial difficulties compared to White patients [12]. Non-White patients with cancer were more likely to report financial hardship than White patients with cancer [1].

There is paucity of studies that focus on healthcare systems where non-medical costs are more common than direct costs. Thus, little information is available on whether cancer survivors with a migration background (defined as *“any person who is moving or has moved across an international border or within a state away from his/her habitual place of residence, regardless of (a) the person’s legal status; (b) whether the movement is voluntary or involuntary; (c) what the causes for the movement are or (d) what the length of the stay is.”* [27]) in Germany, or in similar healthcare systems, experience different levels of financial difficulties compared to non-migrant patients, and how these difficulties evolve over time. However, speculations have been published on a possible association between migration background and financial difficulties. In a series of qualitative interviews, Hempler et al. [28] reported that oncological personnel in Germany considered patients with cancer and migration background to have higher risk to experience financial difficulties due to lower health literacy and reduced ability to navigate the German health system.

### Aim

This study aimed to explore the trajectory of financial difficulties among breast cancer survivors in Germany and whether this trajectory is associated with the migration background of the survivors. We considered the following research questions:

- How does the trajectory of financial difficulties change in breast cancer survivors in Germany?
- Among breast cancer survivors in Germany, is a migration background associated with the trajectory of financial difficulties?

## Study population and methods

### Data collection

Data collection took place in the prospective multicentre cohort study BRENDA II (Breast Cancer under Evidence-Based Guidelines), which was conducted in four certified breast cancer centres (University Medical Centre in Ulm and the Hospitals of Kempten, Memmingen and Esslingen) between 2009 and 2016. Patients with primary breast cancer were included, while patients with (a) metastatic or recurrent disease at baseline, (b) bilateral tumour, (c) primary occult disease or *phylloides* tumour, as well as (d) patients unable to complete a questionnaire, and (e) patients who did not return a written informed consent were excluded [29–33].

Patients were approached four times: before surgery (t1), before the beginning of the adjuvant therapy (t2), at the end of the adjuvant therapy (t3), and five years after surgery (t4). The treating physician informed the patients about the study and, upon agreeing to participation, handed out the questionnaire and conducted the first interview. Follow-up interviews were conducted by trained study nurses [29–33].

Ethical approval was obtained from the Ethics Committee of Ulm University [29–33].

## Operationalization of the variables

*Financial difficulties* was coded as a dichotomous variable (no/yes) from the original item of the European Organisation for Research and Treatment of Cancer Core Instrument (EORTC QLQ-C30) [34] with a cut-off value for *yes* at > 17 points [35]. In order to observe the changes in financial difficulties based on the different events during the trajectory of the disease (e.g. diagnosis, surgery, adjuvant treatment, survivorship), a categorical variable for the *trends in financial difficulties* was created and coded as: (a) *always,* if financial difficulties were present at every time-point, (b) *never,* if financial difficulties were present at no time-point, (c) *initial,* if financial difficulties were present at t1 but not at t4, (d) *delayed,* if financial difficulties were present only at t4, and (e) *acquired*, if present at t2 and/or t3 but not at t1. Records that did not enter in this classification were coded as (f) *other*.

*Migration background* was defined based on the place of birth (t1), country of main residency (t1), citizenship (t1), and nationality (t1) of the participants. Participants were defined as migrants if they were born and/or resided mainly in a country other than Germany and/or possessed citizenship/nationality other than German.

*Severity of the disease* (t1) was considered as a potential confounder. It was coded as a categorical variable (low-to-intermediate risk of death/high risk of death/missing) based on the St. Gallen criteria [36].

*Trends in partnership status* was coded from the values of partnership status (co-inhabiting/non-co-inhabiting with partner/missing) at t1 and t4. The newly created variable was coded as (always co-inhabiting/never co-inhabiting/co-inhabiting yes-to-no/co-inhabiting no-to-yes). Trends in partnership status was considered as a potential effect modifier. Partnership status at t1 was reported in the socio-demographic description of the participants.

Other socio-demographic characteristics of the participants were considered as potential effect modifiers: *age* at t1 (< 45 years/45–65 years/ > 65 years/missing), and/or as potential confounders: e*ducational level* at t1 (≤ 10 years/ > 10 years of education/missing).

*Employment status* at t1 (employed/non-employed/missing) and *monthly equivalent household income* at t1 (< 1,000 euros/between 1,000 and 2,000 euros/ > 2,000 euros/missing) were included for the description of the study sample. Monthly equivalent household income at t1 and t4 was calculated using the mean value of the self-reported monthly household income class and number of persons in the household using the OECD-modified scale [37]. In order to compare the equivalent income at the two time-points, a variable for *trends in equivalent income* (stable/increased/decreased/missing) was created.

*Progression of the disease* at t4 (no progression/recidivism and/or metastases/missing) was also included.

## Statistical Analysis

### Study sample

In order to allow for trend comparisons, this analysis considered the participants who completed the financial difficulties item of the EORTC QLQ-C30 at all four time-points.

### Sample Description

The study population was described in absolute and relative frequencies for migration background, trends in financial difficulties as well as age, education, employment status, monthly equivalent household income, severity of the disease, and partnership status. The socio-demographic variables were also reported by migration background and trends in financial difficulties.

### Outcome analysis

A multinomial logistic regression analysis was conducted. Trends in financial difficulties (always/never/initial/delayed/acquired/other; *reference* = *never*) was considered the outcome of interest, and migration background (yes/no/missing; *reference* = *yes*) was considered the exposure of interest.

Age (≤ 65 years/ > 65 years/missing; *reference* =  ≤ *65 years*) and trends in partnership status (always co-inhabiting/never co-inhabiting/co-inhabiting yes-to-no/co-inhabiting no-to-yes; *reference* = *always co-inhabiting*) were considered as potential effect modifiers. The presence of effect modification was tested with Likelihood-Ratio tests.

Education (≤ 10 years/ > 10 years of education/missing; *reference* =  > *10 years*) and severity of the disease at t1 (low-to-intermediate risk of death/high risk of death/missing; *reference* = *low-to-intermediate risk of death*) were included in the initial model as potential confounders.

The final model was defined via step-wise forward selection of potential confounders. Change ≥ 10% in the estimate of the association between migration background and trends in financial difficulties was considered as the cut-off for confounders to be considered relevant.

Employment status, monthly equivalent household income, and trends in equivalent income were considered as variables on the causal pathway of the association of interest and thus not included in the model.

## Drop-out and missing values analysis

The potential presence of selection bias in the study was explored via drop-out analysis. This analysis compared the socio-demographic characteristics of the population included in this analysis with the population originally included in the BRENDA II study (Table 5). In addition, we reported the absolute numbers of participants who completed the financial difficulties item of the EORTC QLQ-C30 at each- and all-time-points (Supplementary material—Figure S1). In order to evaluate potential patterns in participation, the univariate association between completing the financial difficulties item at each- and all-time-points or not and the socio-demographic characteristics of the participants (migration background, age class, educational level, employment status, monthly equivalent household income, severity of the disease and partnership status) was calculated (Supplementary material—Table S1).

Missing values were reported in their absolute and relative frequencies. No imputation of missing values was conducted.

All analyses were conducted with SAS 9.4 (Statistical Analysis Software 9.4, SAS Institute Inc., Cary, North Carolina, USA).

## Results

### Description of the study sample

*N* = 363 participants completed the financial difficulties item of the EORTC QLQ-C30 at every time-point and were included in this study. A chart representing the study sample at each time-point is included in the supplementary material (Figure S1).

Most of the participants were non-migrant (86%), employed (88%) and co-inhabiting with a partner (77%). Most of the participants had a lower education level (79% with 10 or less years of education) and a low-to-intermediate risk of death from the disease (83%) (Table 1).

**Table 1:** Description of the socio-demographic characteristics and trends in financial difficulties of the study sample (*N* = 363)

Covariates		N	%
Migration background			
No		314	86.5
Yes		48	13.2
Missing		1	0.3
Age class			
Under 45 years		28	7.7
Between 45 and 65 years		216	59.5
Over 65 years		119	32.8
Educational level			
≤ 10 years		288	79.3
> 10 years		73	20.1
Missing		2	0.6
Employment status			
Non-employed*		43	11.9
Employed		320	88.2
Monthly equivalent household income			
< 1,000 euros		119	32.8
Between 1,000 and 2,000 euros		162	44.6
Missing		82	22.6
Trends in equivalent income			
Stable		59	16.2
Increased		6	1.6
Missing		298	82.1
Severity of the disease			
Low-to-intermediate risk of death		301	82.9
High risk of death		60	16.5
Missing		2	0.6
Progression of the disease			
No progression		348	95.9
Recidivism and/or metastases		15	4.1
Partnership status			
Non-co-inhabiting		80	22.0
Co-inhabiting		278	76.6
Missing		5	1.4
Trends in partnership status			
Never co-inhabiting		72	19.8
Always co-inhabiting		213	58.7
Co-inhabiting yes-to-no		65	17.9
Co-inhabiting no-to-yes		8	2.2
Missing		5	1.4
Trends in financial difficulties			
Never		184	50.7
Always		26	7.2
Initial		17	4.7
Delayed		36	9.9
Acquired		78	21.5
Other		22	6.1

*Non-employed includes retired, unemployed, homemaker, in training

### Trends in financial difficulties

**Fig. 1:**
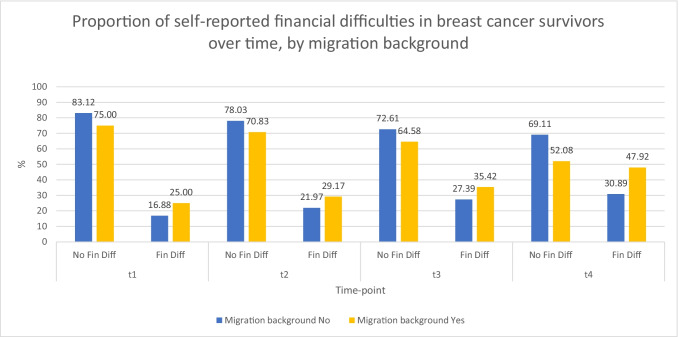
Proportion of the presence or absence of financial difficulties (“Fin diff”) in the overall study sample over all time-points (before surgery [t1], before adjuvant therapy [t2], after adjuvant therapy [t3], and five years from surgery [t4]). Missing values for financial difficulties or migration background are not reported. Presence or absence of financial difficulties (no/yes) was defined from the original item of the European Organisation for Research and Treatment of Cancer Core Instrument (EORTC QLQ-C30) [34], with a cut-off value of > 17 for the presence of financial difficulties, following Giesinger et al. [35]

Half of the participants (51%) reported to have never experienced financial difficulties throughout the disease. Sixty-five participants (18%) reported financial difficulties at t1, 83 (23%) at t2, 103 (28%) at t3, and 120 (46%) at t4. The reported presence of financial difficulties at each time-point stratified by migration background is provided in Figure 1.

Financial difficulties were always present in 26 participants (7%). Initial financial difficulties were present in 17 participants (5%), delayed financial difficulties in 36 participants (10%), and acquired financial difficulties in 78 participants (21%) (Table 1).

Participants younger than 45 years of age more often reported having always experienced financial difficulties and less often having never experienced them (35% vs 3%). Conversely, participants older than 65 years of age more often reported never having experienced financial difficulties and less often having always experienced them (42% vs 8%). Participants not living with a partner more often reported having always experienced financial difficulties and less often having never experienced them (38% vs 18%). Participants with a high risk of death from the disease more often reported having always experienced financial difficulties and less often having never experienced them (31% vs 11%) (Table 2).

**Table 2:** Socio-demographic characteristics of the study sample by trends in financial difficulties (*N* = 363)

Covariates	Trends in financial difficulties											
	Never		Always		Initial		Acquired		Delayed		Other	
	N	%	N	%	N	%	N	%	N	%	N	%
Migration background												
No	168	91.3	22	84.6	14	82.4	64	82.1	29	80.6	17	77.3
Yes	15	8.2	4	15.4	3	17.7	14	18.0	7	19.4	5	22.7
Missing	1	0.5	0	0	0	0	0	0	0	0	0	0
Age class												
Under 45 years	6	3.3	9	34.6	2	11.8	6	7.7	3	8.3	2	9.1
Between 45 and 65 years	101	54.9	15	57.7	8	47.1	55	70.5	21	58.3	16	72.7
Over 65 years	77	41.9	2	7.7	7	41.2	17	21.8	12	33.3	4	18.2
Educational level												
≤ 10 years	43	23.4	7	26.9	2	11.8	12	15.4	3	8.3	6	27.3
> 10 years	140	76.1	18	69.2	15	88.2	66	84.6	33	91.7	16	72.7
Missing	1	0.5	1	3.9	0	0	0	0	0	0	0	0
Employment status												
Non-employed*	24	13.0	3	11.5	3	17.7	6	7.7	5	13.9	2	9.1
Employed	160	87.0	23	88.5	14	82.4	72	92.3	31	86.1	20	90.9
Monthly equivalent household income												
< 1,000 euros	41	22.3	8	30.7	5	29.4	40	51.2	15	41.7	10	22.7
Between 1,000 and 2,000 euros	102	55.4	11	42.3	8	47.1	24	30.8	10	27.8	7	31.8
Missing	41	22.3	7	26.9	4	23.5	14	17.9	11	30.6	5	22.7
Trends in equivalent income												
Stable	25	84.2	6	23.1	5	29.4	11	14.1	7	19.4	5	22.7
Increased	4	13.6	1	3.8	0	0	1	1.3	0	0	0	0
Missing	155	84.2	19	73.1	12	70.6	66	84.6	29	80.6	17	77.3
Severity of the disease												
Low-to-intermediate risk of death	162	88.0	18	69.2	14	82.4	58	74.4	30	83.3	19	86.4
High risk of death	20	10.9	8	30.8	3	17.7	20	25.6	6	16.7	3	13.6
Missing	2	1.1	0	0	0	0	0	0	0	0	0	0
Progression of the disease												
No progression	181	98.4	24	92.3	14	82.4	74	94.9	35	97.2	20	90.9
Recidivism and/or metastases	3	1.6	2	7.7	3	17.7	4	5.1	1	2.8	2	9.1
Partnership status												
Non-co-inhabiting	33	17.9	10	38.5	3	17.7	22	28.2	7	19.4	5	22.7
Co-inhabiting	148	80.4	16	61.5	13	76.5	55	70.5	29	80.6	17	77.3
Missing	3	1.6	0	0	1	5.9	1	1.3	0	0	0	0
Trends in partnership status												
Never co-inhabiting	32	17.4	7	26.9	2	11.8	20	25.6	7	19.4	4	18.2
Always co-inhabiting	112	60.9	12	46.2	8	47.1	47	60.3	23	63.9	11	50.0
Co-inhabiting yes-to-no	36	19.6	4	15.4	5	29.4	8	10.3	6	16.7	6	27.3
Co-inhabiting no-to-yes	1	0.5	3	11.5	1	5.9	2	2.6	0	0	1	4.6
Missing	3	1.6	0	0	1	5.9	1	1.3	0	0	0	0

^*^Non-employed includes retired, unemployed, homemaker, in training

## Comparison of migrant and non-migrant participants

### Socio-demographic characteristics

Compared to non-migrants, survivors with a migration background were more often highly educated (27% vs 19% with > 10 years of education), more often unemployed (17% vs 11%), and reported a monthly equivalent income of less than 1,000 euros more often (50% vs 30%) (Table 3).

**Table 3:** Socio-demographic characteristics and trends in financial difficulties of the study sample by migration background (*N* = 363)

Covariates	Migration background								
		No			Yes			Missing	
		*N*	%		*N*	%		*N*	%
Age class									
Under 45 years		22	7.0		5	10.4		1	100
Between 45 and 65 years		192	61.2		24	50.0		0	0
Over 65 years		100	31.9		19	39.6		0	0
Educational level									
≤ 10 years		253	80.6		35	72.9		0	0
> 10 years		60	19.1		13	27.1		0	0
Missing		1	0.3		0	0		1	100
Employment status									
Non-employed*		35	11.1		8	16.7		0	0
Employed		279	88.9		40	83.3		1	100
Monthly equivalent household income									
< 1,000 euros		95	30.2		24	50.0		0	0
Between 1,000 and 2,000 euros		148	47.1		13	27.1		1	100
Missing		71	22.6		11	22.9		0	0
Trends in equivalent income									
Stable		51	16.2		8	16.7		0	0
Increased		6	1.9		0	0		0	0
Missing		257	81.8		40	83.3		1	100
Severity of the disease									
Low-to-intermediate risk of death		265	84.4		35	72.9		1	100
High risk of death		48	15.3		12	25.0		0	0
Missing		1	0.3		1	2.1		0	0
Progression of the disease									
No progression		303	96.5		44	91.7		1	100
Recidivism and/or metastases		11	3.5		4	8.3		0	0
Partnership status									
Non-co-inhabiting		74	23.6		6	12.5		0	0
Co-inhabiting		237	75.5		40	83.3		1	100
Missing		3	1.0		2	4.2		0	0
Trends in partnership status									
Never co-inhabiting		67	21.3		5	10.4		0	0
Always co-inhabiting		182	58.0		30	62.5		1	100
Co-inhabiting yes-to-no		55	17.5		10	20.8		0	0
Co-inhabiting no-to-yes		7	2.2		1	2.1		0	0
Missing		3	1.0		2	4.2		0	0
Trends in financial difficulties									
Never		168	53.5		15	31.2		1	100
Always		22	7.0		4	8.3		0	0
Initial		14	4.5		3	6.3		0	0
Delayed		29	9.2		7	14.6		0	0
Acquired		64	20.4		14	29.2		0	0
Other		17	5.4		5	10.4		0	0

^*^Non-employed includes retired, unemployed, homemaker, in training

### Trends in financial difficulties

Compared to non-migrants, participants with a migration background reported having never experienced financial difficulties throughout the disease less often (31% vs 53%) and more often to have experienced financial difficulties in the later stages of survivorship (15% vs 9%) (Table 3).

### Financial difficulties in migrants vs. non-migrants

Age and trends in partnership status did not act as effect modifiers for the association of interest, but rather as confounders together with education and thus were all included in the model selection. Due to a negligible confounding effect, severity of the disease did not enter the final model.

Compared to non-migrants, survivors with a migration background were almost four times more likely to have experienced financial difficulties in the very final stages of the follow-up (OR = 3.7; 95% CI 1.3, 10.5) or to acquire difficulties during the disease (OR = 3.6; 95% CI 1.6, 8.4), rather than not experiencing them at all, after adjusting for age, trends in partnership status, and education (Table 4).

**Table 4:** Trends in financial difficulties in breast cancer survivors with versus without a migration background. Displayed are the odds ratios obtained from the multinomial regression analysis after adjusting for age class, trends in partnership status, and educational level. (*N* = 362)

Exposure of interest		Trends in financial difficulties	Odds Ratio	95% Confidence interval		*p*
				Lower	Upper	
		Never	*Reference*			0.02
Migration background (*Reference* = *"Yes"*)		Always	3.2	0.9	11.6	
		Initial	2.4	0.6	10.3	
		Delayed	3.7	1.3	10.5	
		Acquired	3.6	1.6	8.4	
		*Other*	*4.3*	*1.3*	*14.2*	

## Drop-out analysis

Compared to the entire BRENDA II study sample, the one used for this analysis comprised fewer unemployed survivors (28% vs 17% among participants with a migration background and 16% vs 11% among participants without a migration background) and fewer participants who did not live with a partner (18% vs 12% in participants with a migration background and 28% vs 24% in participants without a migration background).

Among survivors with a migration background, we found a higher proportion with a high risk of death from the disease compared to the entire sample (25% vs 22%). Among migrants, there was a higher proportion of survivors with a monthly equivalent income lower than 1,000 euros compared to the original sample (50% vs 41%) (Table 5).

**Table 5:** Drop-out analysis. Socio-demographic characteristics and trends in financial difficulties of the study sample included in this analysis (SA, *N* = 363) and the population originally recruited for the BRENDA II study (PS, *N* = 759), by migration background (values with missing migration background were not reported, PS = 6, SA = 1)

Covariates	Migration background							
	No				Yes			
	PS		SA		PS		SA	
	N	%	N	%	N	%	N	%
Age class (t1)								
Under 45 years	48	7.6	22	7.0	14	11.3	5	10.4
Between 45 and 65 years	351	55.8	192	61.2	63	50.8	24	50.0
Over 65 years	230	36.6	100	31.9	47	37.9	19	39.6
Educational level (t1)								
≤ 10 years	512	81.4	253	80.6	88	71.0	35	72.9
> 10 years	115	18.3	60	19.1	35	28.2	13	27.1
Missing	2	0.3	1	0.3	1	0.8	0	0
Employment status (t1)								
Non-employed*	98	15.6	35	11.1	35	28.2	8	16.7
Employed	531	84.2	279	88.9	89	71.8	40	83.3
Monthly equivalent household income (t1)								
< 1,000 euros	188	29.9	95	30.2	51	41.1	24	50.0
Between 1,000 and 2,000 euros	264	42.0	148	47.1	37	29.8	13	27.1
> 2,000 euros	4	0.6	0	0	0	0	0	0
Missing	173	27.5	71	22.6	36	29.0	11	22.9
Severity of the disease (t1)								
Low-to-intermediate risk of death	529	84.1	265	84.4	95	76.6	35	72.9
High risk of death	95	15.1	48	15.3	27	21.8	12	25.0
Missing	5	0.8	1	0.3	2	1.6	1	2.1
Partnership status (t1)								
Non-co-inhabiting	174	27.7	74	23.6	23	18.5	6	12.5
Co-inhabiting	451	71.7	237	75.5	99	79.8	40	83.3
Missing	4	0.6	3	1.0	2	1.6	2	4.2

^*^Non-employed includes retired, unemployed, homemaker, in training

## Discussion

We aimed to explore the trends of financial difficulties in breast cancer survivors in Germany and their association with migration background.

Compared to non-migrants, cancer survivors with a migration background were almost 4 times more likely to have experienced financial difficulties in the later stages of the follow-up or at some point during the trajectory of the disease. To the knowledge of the authors, this study is the first to explore the association of financial difficulties in breast cancer survivors and their migration background in Germany. Hence, these results are difficult to compare to previous studies on the topic, which took place in healthcare systems different from the one in Germany and focused on ethnic groups rather than on migration background [1, 3]. Following Hempler et al. [28], a higher chance of experiencing financial difficulties for cancer survivors with a migration background might be due to the lack of knowledge of the German health system. The study reported the perception of medical personnel according to whom this lack of knowledge led to untimely planning of financial support or insurance coverages [28]. Even if still on a speculative level, this association between health literacy and planning of financial support was also noted in the work by Jagsi et al. [38], in which the worsening of the financial status of ethnic minority patients compared to White patients was only significant among Spanish-speaking Latina patients, and not among English-speaking Latina or Black patients. These aspects might also be observed in our results. The differences based on migration background were clearer in the later stages of the follow-up, while no significant difference was present at the beginning of the disease. This scenario could potentially be explained by lower knowledge of the health system and a lower ability to plan financial support or coverage.

When interpreting these results, it is important to consider the possible selection bias in the BRENDA II study sample from t1 to t4. In this study, we included a higher proportion of migrant cancer survivors with the lowest income and the highest risk of death due to the disease compared to the ones included in the first time-point of the study. Both low monthly income and health status acted as predictors of financial difficulties in patients with cancer [1, 3]. These differences in proportions were not equally present in the non-migrant population. Thus, this study could suffer from a selection bias towards cancer survivors with a migration background more likely to experience financial difficulties compared to non-migrants. This can be also seen in the univariate association between completing the financial difficulties item of the EORTC QLQ-C30 or not at all-time-points (and—thus—being included in this analysis) and the socio-demographic characteristics of the participants. Significant associations for migration background, age class, and employment status were present (Supplementary information—Table S1).

In terms of the overall prevalence of financial difficulties in cancer survivors in Germany, we observed that 49% of the survivors reported financial difficulties at at least one time-point in the study. This result is in accordance with other studies on the topic: Sharp et al. [39] observed that 49% of patients reported financial difficulties. Zafar et al. [13] observed that 42% of patients with cancer reported a significant financial burden. Essue et al. [2] observed that 44% of the patients reported financial difficulties. Büttner et al. [5] observed that 40% of patients reported financial difficulties three months after leaving the hospital. The slightly higher proportion of financial difficulties in this study could be explained by the fact that the population of this study comprised female survivors only, and female gender has been seen to be a predictor of financial difficulties for cancer survivors [3]. This effect might be balanced following the results of the drop-out analysis: we included higher proportions of employed and co-inhabiting participants compared to the original BRENDA II population. Partnership status and income were associated with lower financial difficulties [1, 3].

## Implications for future research

Further research should focus on understanding why migrant cancer survivors in Germany report higher financial difficulties than non-migrants and whether the specific types of financial difficulties (e.g. medical/non-medical costs, inability to work, etc.) differ based on the ethnical and/or cultural background of the individuals. Moreover, using validated measures, future research should try to understand the role of acculturation (defined as *“culture change that is initiated by the conjunction of two or more autonomous cultural systems. Acculturative change may be the consequence of direct cultural transmission; it may be derived from non-cultural causes, such as ecological or demographic modification induced by an impinging culture; it may be delayed, as with internal adjustments following upon the acceptance of alien traits or patterns; or it may be a reactive adaptation of traditional modes of life”* [40]) and the ability to navigate the health system in the association between survivors with a migration background and financial difficulties after cancer. Finally, the limitations of this study in terms of gender and type of cancer could be overcome.

## Implications for clinical work

Healthcare personnel might have difficulties to engage in financial or work-related discussions with patients and survivors: one occupational doctor in five (19%) felt insufficiently trained to engage in employment-related discussions with the patients [41]. Jagsi et al. [42] observed that 55%, 17%, and 26% of surgeons, medical oncologists, and radiation oncologists, respectively, reported to never or rarely discuss financial difficulties with their patients. Altomare et al. [43] observed that 41% of physicians reported to rarely or never discuss financial matters with the patients. This aspect might be exacerbated when advising patients and survivors with a migration background, as different ethnic and/or cultural groups have different approaches when mentioning financial needs [44], resulting in confusion for the medical personnel and unmet information needs for the patients. Hempler et al. [28] reported that oncologists perceive financial matters to be less central to patients with cancer and a migration background compared to German patients. Jagsi et al. [42] observed that 31%, 30%, and 25% of Black, Latin, and Asian patients, respectively, reported to desire specific consultation with the medical personnel regarding their employment and/or finances, compared to the 15% of White patients. More than half of the patients who reported wanting a specific consultation (55%) reported not having had a relevant discussion on the financial impact of the disease, and for almost three-quarters (73%) of the patients who reported financial worries, the medical personnel was not of help [42].

Therefore, in the light of our results, medical personnel might consider to ask more actively patients and survivors about their possible financial difficulties, especially the ones with a migration background. Informational material regarding support services and insurances should be available, ideally in multiple languages.

## Limitations

Besides the aforementioned limitations in terms of selection and composition of the study sample, we assessed financial difficulties with a single item as part of a questionnaire. As any self-reported item, it could suffer from information bias and it does not represent a measure of sustained costs but rather of the perception of the disease-induced financial burden, which might be influenced by the presence of a psychological financial difficulty rather than a material difficulty [1]. The process of selecting only participants with complete data (we considered only participants who answered at all time-points) for the present analysis might also have introduced bias. The exclusion of records with missing values might have favoured participants with specific characteristics, e.g. lower symptom burden, lower risk of death from the disease or from old age, lower shame in reporting financial difficulties, and a higher ability to understand the question (and therefore higher education and/or German proficiency). These characteristics predict lower financial difficulties [1, 3, 12]. Hence, the results of this study could be underrepresenting the financial difficulties reported in the original BRENDA II study sample.

The migration background status of the participants was built ad hoc for this study: the variable does not retain any information on the level of acculturation of the survivors nor on their ability to navigate the German health system. Moreover, the proportion of migrants in the study population is small (13%). For this reason, it was also not possible to conduct a stratified analysis based on the country of origin of the participants, which could have—even if only descriptively—highlighted different associations with financial difficulties between the different migrant groups.

This study comprised only one gender and one type of cancer. Both female gender and breast cancer could directly or indirectly (e.g. return to work) influence the perception of financial difficulties in the participants. Thus, results should be generalized only to female breast cancer survivors.

## Conclusions

Half of female breast cancer survivors in Germany experience financial difficulties at some time-point in the five years following the first surgery. Cancer survivors with migration background are almost four times more likely than non-migrant survivors to experience these difficulties, especially in the later stages of the follow-up. This study represents a first exploration of cancer-related financial difficulties based on the migration background of cancer survivors in Germany. However, due to the paucity of similar research and the limitations already mentioned, the results should be generalized considering the specific composition of the study population in terms of gender and cancer type.

## Supplementary Information

Below is the link to the electronic supplementary material.

Supplementary file1 (DOCX 47 KB)

## Data Availability

Not applicable.

## Code availability

Code is available upon request to the corresponding author.
