# Supplementary material for: Financial difficulties in breast cancer survivors with and without migration background in Germany—results from the prospective multicentre cohort study BRENDA II
Source: Support Care Cancer. 2022 May 4;30(8):6677–88. doi: 10.1007/s00520-022-07074-7 (PMC9213307; doi:10.1007/s00520-022-07074-7)
Supplement: Supplementary file 1 — Supplementary file1 (DOCX 47 KB) [file 520_2022_7074_MOESM1_ESM.docx]

# Supplementary information

*N* = 759 took part in BRENDA II t1

Took part in the Brenda II study

t1 – Before surgery

*N* = 707 (16% with migration background)

Completed the financial difficulties item of the EORTC QLQ-C30 at the specific time-point

t2 – Avg. one month after surgery

*N* = 648 (17% with migration background)

t3 – Avg. 8 months after surgery

*N* = 626 (17% with migration background)

t4 – Five years after surgery

*N* = 452 (14% with migration background)

Included in the analysis

*N* = 363 (13% with migration background)

Completed the financial difficulties item of the EORTC QLQ-C30 at each time-point

**Figure S1**. Absolute numbers of the participants in the BRENDA – II study who completed the financial difficulties item of the European Organisation for Research and Treatment of Cancer Core Instrument (EORTC QLQ-C30) at each and at all-time points. In brackets are reported the proportion of participants with a migration background.

| **Table S1**. Univariate association between completing or not the financial difficulties item of the European Organisation for Research and Treatment of Cancer Core Instrument (EORTC QLQ-C30) at each time point and the socio-demographic characteristics of the participants. The participants/non-participants at each time point as well as this analysis are compared to the original *N* = 759 patients included in the BRENDA II study at t1. The total number of people completing the questionnaire or being included are reported in brackets after each corresponding time-point. Reported are the *p*-values of the Wald Chi-squared test. | | | | | |
| --- | --- | --- | --- | --- | --- |
| Potential predictors | t1 (707) | t2 (648) | t3 (626) | t4 (452) | Analysis (363) |
|  | *p* | *p* | *p* | *p* | *p* |
| Migration background | 0.0359 | 0.3649 | 0.5589 | 0.0292 | 0.0231 |
| Age class | 0.0033 | 0.8662 | 0.2124 | 0.0004 | 0.0310 |
| Educational level | <.0001 | 0.7720 | 0.0270 | 0.6732 | 0.3392 |
| Employment status | 0.1279 | 0.9038 | 0.9391 | <.0001 | 0.0001 |
| Monthly equivalent household income* | 0.0344 | 0.6887 | 0.1136 | 0.1119 | 0.6727 |
| Severity of the disease** | 0.1872 | 0.4778 | 0.2529 | 0.5665 | 0.7764 |
| Partnership status | 0.0002 | 0.4757 | 0.2337 | 0.0232 | 0.0599 |
| **Calculated using the mean value of the self-reported monthly household income class and number of persons in the household using the OECD-modified scale*  ***Coded as categorical variable (low-medium risk of death/high risk of death/missing) based on St. Gallen criteria* | | | | |  |
